# Leveraging continuous glucose monitoring for personalized modeling of insulin-regulated glucose metabolism

**DOI:** 10.1038/s41598-024-58703-6

**Published:** 2024-04-05

**Authors:** Balázs Erdős, Shauna D. O’Donovan, Michiel E. Adriaens, Anouk Gijbels, Inez Trouwborst, Kelly M. Jardon, Gijs H. Goossens, Lydia A. Afman, Ellen E. Blaak, Natal A. W. van Riel, Ilja C. W. Arts

**Affiliations:** 1 Maastricht Centre for Systems Biology (MaCSBio), Maastricht University, Maastricht, The Netherlands; 2 Department of Data Science and Knowledge Discovery, Simula Metropolitan Center for Digital Engineering, Oslo, Norway; 3 Department of Biomedical Engineering, Eindhoven University of Technology, Eindhoven, The Netherlands; 4 Division of Human Nutrition and Health, Wageningen University, Wageningen, The Netherlands; 5 Department of Human Biology, NUTRIM School of Nutrition and Translational Research in Metabolism, Maastricht University Medical Center, Maastricht, The Netherlands

**Keywords:** Type 2 diabetes, Pre-diabetes, Dynamical systems, Computational models

## Abstract

Continuous glucose monitoring (CGM) is a promising, minimally invasive alternative to plasma glucose measurements for calibrating physiology-based mathematical models of insulin-regulated glucose metabolism, reducing the reliance on in-clinic measurements. However, the use of CGM glucose, particularly in combination with insulin measurements, to develop personalized models of glucose regulation remains unexplored. Here, we simultaneously measured interstitial glucose concentrations using CGM as well as plasma glucose and insulin concentrations during an oral glucose tolerance test (OGTT) in individuals with overweight or obesity to calibrate personalized models of glucose-insulin dynamics. We compared the use of interstitial glucose with plasma glucose in model calibration, and evaluated the effects on model fit, identifiability, and model parameters’ association with clinically relevant metabolic indicators. Models calibrated on both plasma and interstitial glucose resulted in good model fit, and the parameter estimates associated with metabolic indicators such as insulin sensitivity measures in both cases. Moreover, practical identifiability of model parameters was improved in models estimated on CGM glucose compared to plasma glucose. Together these results suggest that CGM glucose may be considered as a minimally invasive alternative to plasma glucose measurements in model calibration to quantify the dynamics of glucose regulation.

## Introduction

The development of type 2 diabetes (T2D) is characterized by accruing deteriorations in the tightly regulated mechanisms of insulin action and insulin secretion, culminating in a loss of glycemic control and hyperglycemia^1^. Assessment of the impaired glucose homeostasis is predominantly based on average glycated hemoglobin (HbA1c), a measure of long-term glycemic control, fasting glucose, or 2h glucose concentrations after an oral glucose tolerance test (OGTT)^2,3^. However, additional assessment of postprandial insulin concentrations provides further insight into (impairments in) insulin sensitivity and secretion^4^.

The hyperinsulinemic-euglycemic clamp, the gold-standard method to quantify insulin sensitivity, is invasive, labor intensive, and does not fully represent normal physiology due to a constant insulin infusion^5^. Therefore, a variety of surrogate measures of insulin sensitivity and $\beta$-cell function have been used, based on fasting and/or OGTT-derived measurements^6–10^. While these surrogate indices may capture particular aspects of glucose regulation, they rely on single time-point or average glucose and insulin values derived from dynamic postprandial data. Hence, the temporal dynamics and the interaction of glucose and insulin responses are not taken into account, potentially masking inter-individual differences in glucose and insulin dynamics.

Physiology-based mathematical models (PBMMs) of glucose regulation can capture the temporal dynamics of glucose regulation, while accounting for the interaction of glucose and insulin^11–13^. Such models have been successfully developed to quantify insulin sensitivity, insulin secretion, and beyond from plasma glucose and insulin concentrations during an OGTT or mixed meal challenge^14–16^. Recently, we have shown that the E-DES model, a PBMM of the insulin-mediated glucose homeostasis was able to capture the heterogeneity in insulin secretion and insulin sensitivity in a large population of individuals with overweight or obesity^17^. However, such model-based quantification of glucose regulation depends on the availability of plasma measurements from clinical experiments, for example an OGTT, to facilitate model calibration. Extending model calibration to include at-home measurements made in a free-living setting may enable the use of PBMMs in digital twin technologies that go beyond their current use in research^18^.

Continuous glucose monitoring (CGM) is gaining popularity in both research and clinical applications, providing information on both short-term patterns (e.g. meal responses) as well as daily or even weekly measures of glycemic variability^19^. The minimally invasive nature, frequent sampling, and potential for real-time monitoring are increasingly exploited for diabetes prevention, management, and beyond^20,21^. The assimilation of CGM data into glucose-insulin models has the potential to broaden the use and impact of PBMMs from in-clinic to free-living conditions and may enable digital twin technologies for the prediction of disease and the optimization of treatment in a more personalized setting^22^. Nevertheless, the use of CGM data necessitates additional considerations such as the lag time of interstitial glucose compared to plasma glucose, as well as the accuracy and reliability of sensor data^23–26^.

Recently, glucose homeostasis models have been fitted to CGM glucose to capture features of glucose responses in healthy individuals, individuals with pre-diabetes, and patients with T2D, and to predict insulin levels in patients with type 1 diabetes (T1D)^27–29^. However, these studies are based on glucose data only, thereby largely ignoring the interaction with insulin. Furthermore, a systematic study of using interstitial glucose from CGM devices compared to the conventional plasma glucose from OGTT for the calibration of PBMMs of glucose-insulin dynamics has yet to be performed.

The aim of the present study was to determine the effect of replacing the conventional plasma glucose measurements with interstitial glucose from CGM devices in the calibration of personalized glucose-insulin models. We made use of simultaneously measured plasma glucose and interstitial glucose data from an OGTT in a population of individuals with overweight or obesity. We compare the calibrated models in terms of model fit, practical identifiability, as well as the association of estimated model parameters with clinically relevant indices of metabolic health.

## Methods

### Data

Data from the PERSonalized Glucose Optimization Through Nutritional Intervention (PERSON) study, a two-centre, randomized, dietary intervention trial was used in this work^30^. The study was performed in line with the principles of the Declaration of Helsinki, and approved by the Medical Ethical Committee of the MUMC+ (NL63768.068.17), and registered at ClinicalTrials.gov (NCT03708419). All participants gave written informed consent. The study design and methodology have been described in detail previously^31^. Inclusion criteria were: age 40–75 years, BMI 25-40 $\hbox {kg/m}^{2}$, body weight stability for at least 3 months (no weight gain or loss >3 kg), and tissue-specific insulin resistance, characterized by predominant muscle or liver insulin resistance. Exclusion criteria included pre-diagnosis of type 2 diabetes, diseases or medication use that affect glucose or lipid metabolism, major gastrointestinal disorders, history of major abdominal surgery, uncontrolled hypertension, smoking, alcohol consumption >14 units/week, and >4 h/week moderate-to-vigorous physical activity. In the week before the start of the dietary intervention (baseline; CIW1) and in the last week (follow-up; CIW2) of the 12-week intervention trial, participants underwent a clinical investigation week.

#### Oral glucose tolerance test

During the clinical investigation week at baseline and 12-week follow-up participants underwent a 7-point oral glucose tolerance test (OGTT) following an overnight fast. Two hundred ml of ready-to-use 75 g glucose solution (Novolab) was ingested within 5 min. Blood samples were collected from the antecubital vein via an intravenous cannula under fasting conditions (t = 0 min) and after ingestion of the glucose drink (t = 15, 30, 45, 60, 90, and 120 min) for determination of plasma glucose and insulin concentrations^30^. Responses with more than two missing samples or missing samples at baseline (t = 0 min) or 2-hour post-load were excluded from the analysis.

#### Continuous glucose monitoring

During the clinical investigation weeks, study participants wore a CGM device for 6 days, including the duration of the OGTT. The CGM device (iPro2 and Enlite Glucose Sensor; Medtronic, Tolochenaz, Switzerland) was worn lateral to the umbilicus and recorded subcutaneous interstitial glucose values every 5 minutes. Participants were asked to perform four daily capillary glucose self-measurements (SMBG) via Contour XT (Ascensia Diabetes Care, Mijdrecht, the Netherlands) while wearing the CGM device. The CGM measurements were then calibrated using the SMBG values in CareLink (Medtronic, Tolochenaz, Switzerland) according to the manufacturer’s instructions. To avoid insufficient calibration, sensor glucose readings outside the time interval of the first and last SMBG measurements were excluded from the analysis. Participants were blinded to the CGM recording, but not to the SMBG values. In addition, CGM data files with irregular measurement frequencies (i.e. other than 5 minute) were excluded from the analysis (n = 3). In order to use the CGM measurements in model calibration, the segment of sensor glucose time-course overlapping with the time of the OGTT test were extracted. Note that the exact sample times of the OGTT were used in this work. Therefore, due to variability in sampling time the time-course may be slightly longer or shorter than the intended 120 minutes.

In total, 404 glucose and insulin responses (228 at baseline and 176 at follow-up) from 237 study participants were included in the analysis. Responses with a missing set of glucose measurements (OGTT, CGM or both) were excluded from the analysis.

#### Metabolic indicators

Indicators of insulin sensitivity including the Matsuda index, HOMA-IR, the area under the glucose curve between baseline and 30 min of the OGTT ($AUC^{30}_{glu}$), the muscle insulin sensitivity index (MISI) and the hepatic insulin resistance index (HIRI) were calculated as previously described^6–9^. A subset (n=76 and n=61 at CIW1 and CIW2, respectively) of the study participants also underwent a 2.5h two-step hyperinsulinemic-euglycemic clamp with constant 40 $mU/m^2/min$ infusion of insulin^31^. The M-value representing peripheral insulin sensitivity was calculated as previously described^5^. Furthermore, indicators of $\beta$-cell function and/or insulin secretion including the disposition index, HOMA-$\beta$, the area under the insulin curve between baseline and 30 min of the OGTT ($AUC^{30}_{ins}$), and the insulinogenic index were also derived as previously reported^30^. The definition of the metabolic indicators used in this work are also described in the Supplementary Appendix.

### E-DES model

The Eindhoven-Diabetes Education Simulator is a physiology-based mathematical model of the human insulin-mediated glucose regulatory system in health, type 1, and type 2 diabetes^32^. The model consists of a gut and plasma compartments within which the change in glucose mass and glucose/insulin concentration over time is described according to coupled differential equations. In addition, we implemented an interstitial compartment describing the diffusion process of glucose from plasma to the interstitial space, as previously employed by Faggionato et al.^33^:

1 $$\begin{aligned} \frac{dG^{i}}{dt} = \frac{1}{\tau _g} (G^{pl}-G^i) \end{aligned}$$

where $G^{pl}$ and $G^{i}$ are glucose concentration in plasma and in interstitium and $\tau _g$ is the equilibration time constant between plasma and interstitium. The interstitial compartment enables the use of interstitial glucose concentrations from CGM devices to be used in model calibration. A complete description of the model can be found in the Supplementary Appendix.

Here, we make use of an implementation with improved computational efficiency. The model source code (using DifferentialEquations.jl^34^) and analysis are available at https://github.com/blzserdos/edes_cgm. The model can simulate the plasma glucose and insulin response of healthy individuals, those with overweight or obesity, individuals with pre-diabetes, or patients with T2D^14,17,35^. Personalized models may be generated by calibrating the model on subject-specific time-series of plasma glucose and insulin concentrations^17^.

### Model calibration

Model parameters representing the rate of glucose appearance in the gut ($k_1$), rate of insulin-dependent glucose uptake to peripheral tissues ($k_5$), and rate of insulin secretion proportional to the elevation in plasma glucose from basal levels ($k_6$, for brevity, referred to as rate of glucose-dependent insulin secretion) were estimated from experimental data. In addition, the lag time ($\tau _g$) was estimated in the case where interstitial glucose is used in calibration. The remainder of the parameters were fixed to population average/median values as previously described in Ref.^17^. The complete set of model parameters including the fixed parameters and constants and their values are listed in the supplement. The experimental data used for calibration consists of a set of simultaneously measured time-series of plasma glucose and insulin concentrations (at t = 15, 30, 45, 60, 90, 120 min) as well as interstitial glucose concentrations from CGM (at t = 5, 10, ..., 120, 125, 130 min) after an OGTT. The t = 0 samples are provided as inputs to the model. For each response, two models were calibrated: one using plasma glucose and plasma insulin, and a second using interstitial glucose and plasma insulin. In other words, the insulin measurements were the same, only the glucose data source was changed between the two models. For brevity, we refer to the data used in model calibration as ’plasma glucose’ in the case of using plasma glucose and plasma insulin measurements, and ’CGM glucose’ in the case of interstitial glucose from CGM device and plasma insulin. The objective in model calibration is

2 $$\begin{aligned} L(\theta ) = \sum ^M_{i=1}\sum ^{N_i}_{j=1}\left( \frac{y_{i,j}-{\hat{y}}_{i,j}({{\varvec{{\theta }}}})}{max(\textbf{y}_i)}\right) ^2 \end{aligned}$$

where *M*, and $N_i$ represent the number of metabolites, and the number of measurement time-points, respectively. The measured data point of metabolite *i* at time-point *j* is denoted by $y_{i,j}$, while $\hat{y_{i,j}}$ is the corresponding model prediction given the parameters ${{\varvec{\theta }}}$. The difference between measurement and prediction is weighted by the maximum of the measured data points to account for the difference in scales between metabolite values. The TikTak multistart optimization algorithm (implemented in MultistartOptimization.jl) was used to optimize the objective in Eq. (2)^36^. The parameter search ranges were constrained to avoid non-physiological parameter configurations. In order to evaluate whether the plasma and CGM measurements are sufficiently informative to determine the model parameters with adequate precision, practical identifiability was assessed via profile likelihood analysis using LikelihoodProfiler.jl^37,38^. We considered the combination of model and data as practically identifiable if the confidence interval of all estimated parameters were of finite size in the parameter scan range^39^. In addition, to highlight the uncertainty in the model estimated observables, profile likelihood-based confidence bands are plotted alongside model simulation in graphics. The confidence bands are derived by estimating confidence intervals (with confidence level 0.95) of an observable (glucose or insulin) as a function of all parameters over simulation time-points^37^. Details of the parameter search, including the search ranges, and profile likelihood analysis are reported in the Supplementary Methods.

Model fit, practical identifiability, and parameter estimates are compared between models calibrated on CGM glucose compared to plasma glucose after an OGTT. The experimental data contains responses at both baseline and follow-up of the PERSON study, however, the interpretation of the intervention effect has been previously reported, and is out of scope for the current study^30^.

## Results

**Figure 1:**
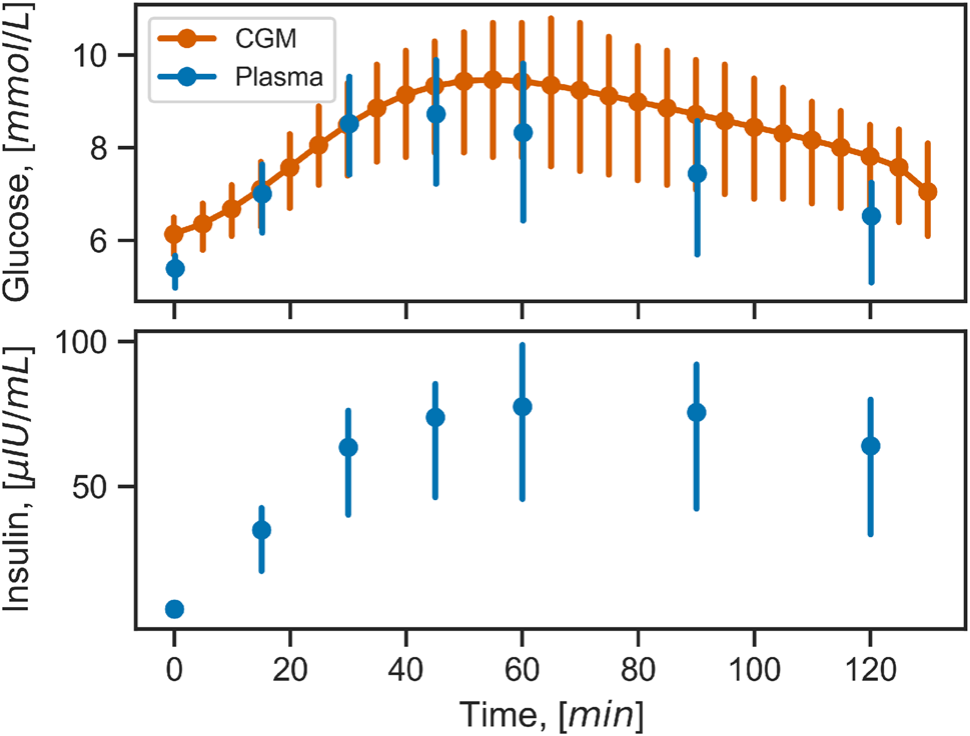
Median glucose and insulin response to an OGTT in individuals with overweight or obesity from the PERSON study. Plasma glucose and interstitial glucose concentrations from CGM devices are shown in the top panel in blue and orange, respectively. Plasma insulin concentration is shown in the bottom panel. Error bars represent the interquartile range. *OGTT* oral glucose tolerance test, *CGM* continuous glucose monitoring.

In total, 404 glucose and insulin responses (228 at baseline and 176 at follow-up) from 237 study participants were included in the analysis. The average glucose and insulin responses to the OGTT are shown in Fig. 1. In general, interstitial glucose was higher than plasma glucose, and the response profiles show a delay and slower dynamics in interstitial compared to plasma glucose.

### Comparison of model fit and diagnostics between models estimated on plasma and CGM glucose

**Figure 2:**
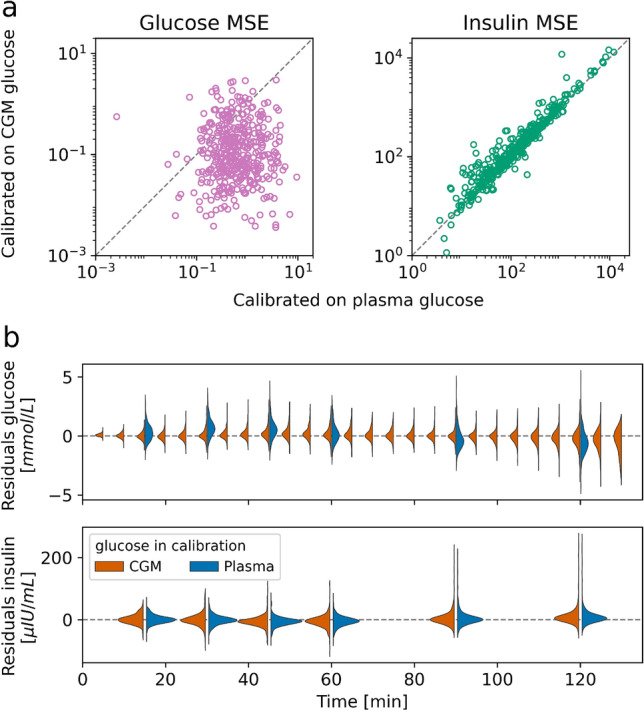
Panel (**a**) Mean squared error (MSE) in the glucose and insulin simulation of the personalized models. Both horizontal and vertical axes use logarithmic scale to better visualize the heavily right skewed error distributions. Panel (**b**) probability density of residuals in glucose and insulin simulation per measurement time-point.

Personalized E-DES models were estimated for each participant based on the plasma glucose and insulin responses. Thereafter, the calibration was repeated with interstitial glucose (’CGM glucose’) instead of plasma glucose leading to a total of 404 personalized models per glucose data type. The model calibration produced parameter estimates representing the rate of glucose appearance in the gut ($k_1$), rate of insulin-dependent glucose uptake to tissues ($k_5$), and rate of glucose-dependent insulin secretion ($k_6$) in each of the personalized models. In addition, $\tau_g$ was estimated in the case of CGM glucose. The mean squared error (MSE) between model simulation and measured data point is shown in Fig. 2, panel a, while the residuals per time point are shown in Fig. 2, panel b. The MSE in the simulation of glucose was higher, indicating a worse fit, when calibrated on plasma glucose compared to CGM glucose. Additionally, the MSE of the personalized models calibrated on plasma and CGM glucose did not correlate well, indicating that some models fitted one or the other glucose data type better (Fig. 2, panel a). In the case of insulin simulation, better agreement was observed between the calibrated models, with slightly higher MSE in models calibrated on CGM glucose. The residuals by measurement time showed that in general the E-DES model captured the trend in the data well, both in the case of plasma glucose and CGM glucose (panel b). The residuals in insulin simulation indicate that few bad model fits were characterized by underestimated insulin concentrations, particularly at the later stage (90, 120 min) of the response.

**Figure 3:**
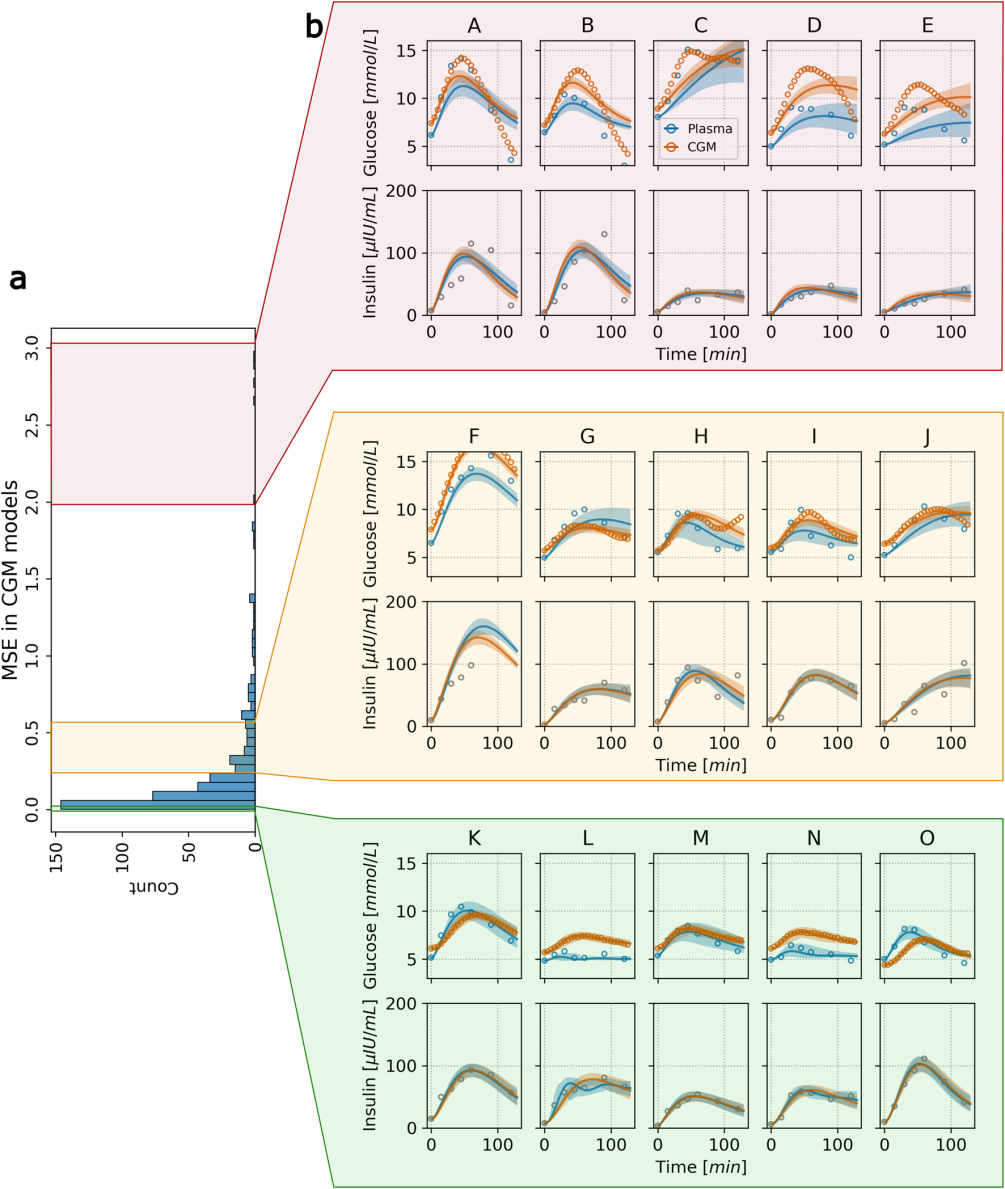
Model fit examples of personalized E-DES models calibrated on CGM glucose, arranged by MSE in the simulation of CGM glucose. Panel (**a**) histogram of MSE in glucose simulation. The three sections with red, yellow, and green shading indicate models with the worst, examples around the mean, and best fit, respectively, as measured by the MSE. Panel (**b**) Glucose and insulin data of participants are shown as circles (A–O) with corresponding model simulation shown as continuous line. Blue and orange color indicates the type of glucose measurement (plasma vs CGM glucose), and simulations from the correspondingly calibrated models. The shaded region around the simulation corresponds to confidence bands generated from estimated confidence intervals (with confidence level 0.95) as function of all parameters over simulation time-points. *CGM* continuous glucose monitoring, *MSE* Mean squared error.

Examples of model fit to experimental data are visualized in Fig. 3. In panel a, the distribution of mean squared error in glucose simulation of personalized models fitted to CGM glucose is displayed as an indicator of model fit. The distribution of MSE is heavily right-skewed, indicating that the majority of models displayed good agreement with the experimental data. There were only 9 out of 404 models with an MSE in CGM glucose above 1.5. Models with the highest MSE (A-E; red shading) and lowest MSE (K-O; green shading) are shown in panel b. The median MSE across the individual models was 0.11. Five randomly selected models in the proximity of the mean MSE (0.27) are shown on F-J (yellow shading). The MSE in plasma glucose showed a similar distribution to that of MSE in CGM data. For completeness, visualization of model fit of personalized models ordered by MSE after calibration on plasma glucose are shown in Supplementary Fig. S3.

Practical identifiability of the personalized models was assessed by profile likelihood analysis (PLA) of the estimated parameters $k_1$, $k_5$, $k_6$ within each calibrated model. As the parameter $\tau_g$ is only meaningful in the case of interstitial glucose and is solely there to account for the delay between plasma and interstitial glucose, we do not include it in PLA. Eleven out of 404 personalized models contained parameters that were practically unidentifiable in the case of calibration based on plasma glucose, while only 6 out of 404 models contained unidentifiable parameters in the case of calibration based on CGM glucose. In all but one of the cases, the unidentifiable parameter was the rate of glucose-dependent insulin secretion ($k_6$).

### Parameter estimates from model calibration on plasma compared to CGM glucose

**Figure 4:**
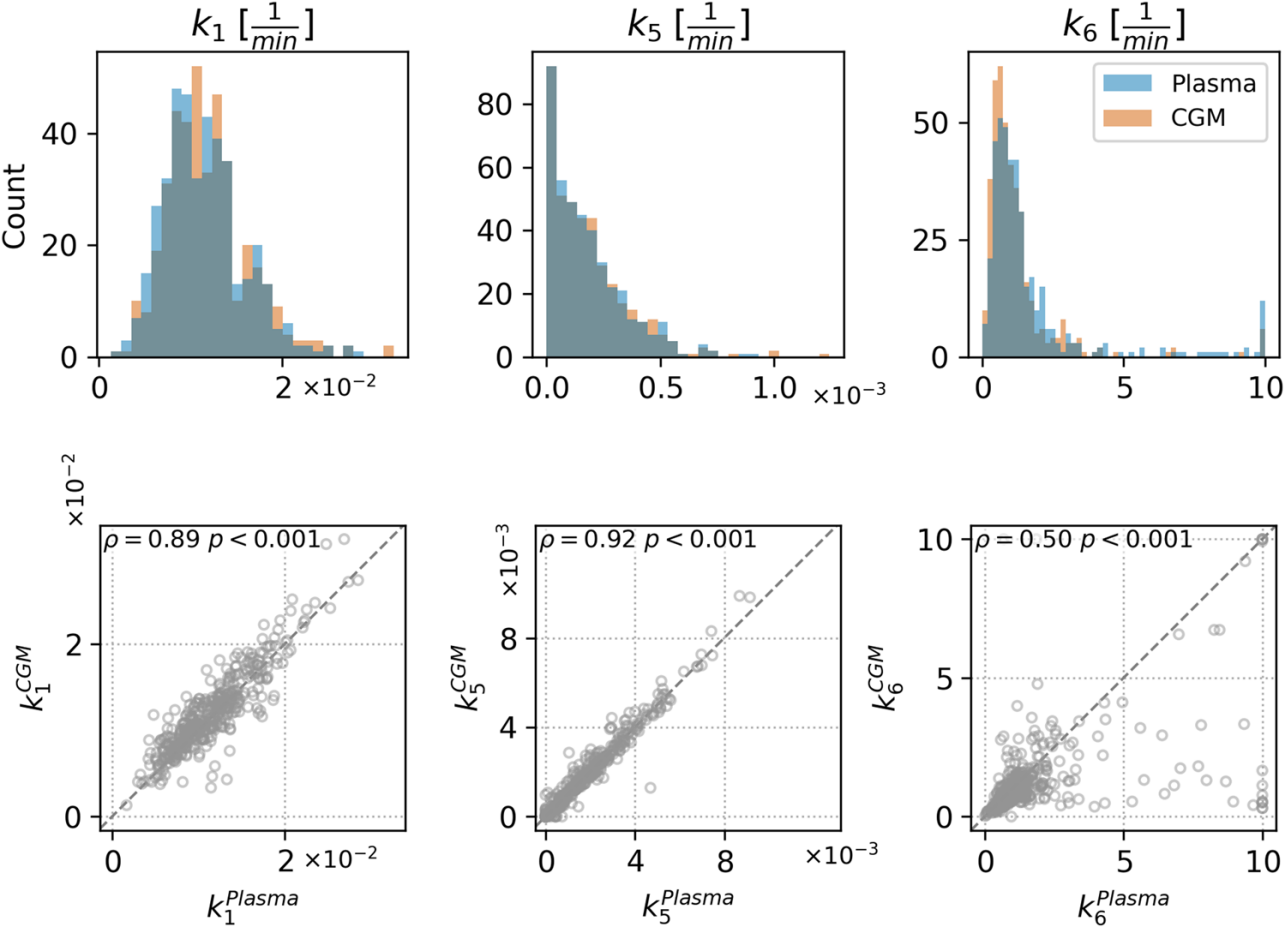
Personalized model parameter estimates resulting from calibration on plasma glucose compared to CGM glucose. Top row: distribution of personalized model parameter estimates. $k_1$: rate of glucose appearance in the gut, $k_5$: rate of insulin-dependent glucose uptake to peripheral tissues, and $k_6$: rate of glucose-dependent insulin secretion. Bottom row: association of parameter estimates estimated from plasma glucose and CGM glucose. Spearman’s $\rho$ is indicated in the top left corner of each panel.

The distribution of parameter estimates and the association between parameters estimated from plasma glucose compared to CGM glucose are shown in Fig. 4. In general, the parameter estimates corresponding to the rate of glucose appearance in the gut ($k_1$) and rate of insulin-dependent glucose uptake to peripheral tissues ($k_5$) showed very good agreement between the models calibrated on plasma compared to CGM glucose (Spearman’s $\rho =0.89, p < 0.001$ for $k_1$, and $\rho =0.92, p < 0.001$ for $k_5$, respectively; Fig. 4, bottom row). No difference in the parameter estimates for $k_1$ and $k_5$ were found between the models estimated on plasma compared to CGM glucose (two-sample Kolmogorov-Smirnov statistic $D=0.073, p=0.22$ and $D=0.027, p=0.99$, respectively). The parameter estimates of $k_6$ showed moderate association (Spearman’s $\rho =0.50, p < 0.001$), and differed between the models calibrated on plasma compared to CGM glucose (Kolmogorov-Smirnov statistic $D=0.12, p < 0.006$). The median $k_6$ estimates were 17.9% higher when calibrated on plasma glucose compared to CGM glucose. In particular, for a small number of responses (N=11 and N=5 in the case of models estimated from plasma and CGM glucose, respectively) the model calibration procedure resulted in $k_6$ estimates on the upper boundary of the parameter search range (Fig. 4, right column). An example of such a model fit is shown in Fig. S3, panel b, sub-panel A, response A. In this case, while the estimated value of $k_6$ is on the upper boundary of the parameter search (10.0), the model fails to capture the glucose response. In order to evaluate whether the difference in the distribution of $k_6$ between the glucose data types was driven by failed model fit associated with parameter estimates on the upper boundary of the $k_6$ search range, we repeated the tests after excluding models (N=16) with $k_6$ estimates on the upper boundary ($k_6 = 10.0$) of the parameter search range. The difference in the distribution of $k_6$ estimates is not statistically significant (Kolmogorov-Smirnov statistic $D=0.095, p=0.051$) after excluding these models. Removing the pairs of personalized models with $k_6$ estimates on the upper bound of the parameter search range results in a Spearman correlation between the plasma and CGM glucose-based estimates of $\rho =0.58, p < 0.001$. Finally, when calibrating on CGM glucose, the median lag time ($\tau _g$) was 2.5 min (with interquartile range $3.7e^{-6} - 8.7$; Fig. S6).

### Parameter estimates association with indicators of glucose homeostasis

Results of the comparison of parameter estimates ($k_1$ and $k_5$ in particular) suggest that the representation of glucose homeostasis between the personalized models obtained via calibration on plasma and CGM data are similar. However, while the general trend shows good agreement, the heterogeneity between plasma and CGM glucose-based personalized models in Fig. 4, in particular in the case of $k_6$, implies that there may be differences in what the models represent between the glucose data types used in calibration. Therefore, we compared the parameter estimates with well-established measures of metabolic health. The models (N=16) with $k_6$ estimates on the upper boundary of the search range were excluded from the analysis. The Spearman correlation of the personalized model parameter estimates and markers of insulin secretion (insulinogenic index, HOMA-$\beta$, AUC$^{30}_{ins}$, disposition index) and insulin sensitivity (Matsuda index, HOMA-IR, MISI, HIRI, AUC$^{30}_{glu}$) including the M-value of the hyperinsulinemic-euglycemic, the gold standard for quantifying peripheral insulin sensitivity are shown in Table 1.

In general, the parameter estimates’ associations with metabolic indicators showed similar trends between models based on plasma glucose compared to CGM glucose. The parameter corresponding to insulin-dependent glucose uptake to periphery ($k_5$) strongly associated with the Matsuda index both when calibrated on plasma and CGM glucose (Spearman’s $\rho =0.75$ and 0.77 both $p < 0.001$, respectively). $k_5$ also showed a moderate association with the disposition index (Spearman’s $\rho =0.65$ and 0.65 both $p < 0.001$, respectively), the M-value (Spearman’s $\rho =0.51$ and 0.51 both $p < 0.001$, respectively), HOMA-IR (Spearman’s $\rho =-0.55$ and $-0.56$ both $p < 0.001$, respectively) and MISI (Spearman’s $\rho =0.50$ and 0.50 both $p < 0.001$, respectively).

Differences between association of metabolic indicators with parameter estimates calibrated on plasma compared to CGM glucose include weaker association of $k_1$ with HIRI (Spearman’s $\rho =0.64$ vs 0.52 both $p < 0.001$, respectively), AUC$^{30}_{ins}$ (Spearman’s $\rho =0.60$ vs 0.50 both $p < 0.001$, respectively), AUC$^{30}_{glu}$ (Spearman’s $\rho =0.40$ vs 0.29 both $p < 0.001$, respectively). In addition, a stronger associations was found when calibrated on CGM glucose compared to plasma glucose in terms of $k_6$ and HIRI (Spearman’s $\rho =0.36$ vs 0.47 both $p < 0.001$, respectively) as well as $k_6$ and AUC$^{30}_{ins}$ (Spearman’s $\rho =0.49$ and 0.56 both $p < 0.001$, respectively). Data are shown in Supplementary Figs. S4 and S5.

**Table 1:** Association of personalized model parameter estimates and indicators of metabolic health.

Metabolic indicator	Spearman’s $\rho$					
	$k_1^{Plasma}$	$k_1^{CGM}$	$k_5^{Plasma}$	$k_5^{CGM}$	$k_6^{Plasma}$	$k_6^{CGM}$
MISI	**0**.**17**	**0**.**18**	**0**.**50**	**0**.**50**	$-$ **0**.**19**	$-$ **0**.**26**
HIRI	**0**.**64**	**0**.**52**	$-$ **0**.**13**	$-$ **0**.**16**	**0**.**36**	**0**.**47**
HOMA-IR	0.03	-0.03	$-$ **0**.**55**	$-$ **0**.**56**	− 0.01	**0**.**13**
Matsuda	− 0.07	0.01	**0**.**75**	**0**.**77**	0.02	$-$ **0**.**17**
$AUC^{30}_{glu}$	**0**.**40**	**0**.**29**	$-$ **0**.**27**	$-$ **0**.**32**	$-$ **0**.**23**	− 0.04
M-value	0.07	0.07	**0**.**51**	**0**.**51**	− 0.04	− 0.07
HOMA-$\beta$	0.04	0.02	$-$ **0**.**29**	$-$ **0**.**27**	**0**.**22**	**0**.**28**
$AUC^{30}_{ins}$	**0**.**60**	**0**.**50**	− 0.06	− 0.09	**0**.**49**	**0**.**56**
insulinogenic index	**0**.**52**	**0**.**45**	0.02	0.00	**0**.**60**	**0**.**61**
disposition index	**0**.**37**	**0**.**38**	**0**.**65**	**0**.**65**	**0**.**51**	**0**.**35**

Bold indicates significance ($p < 0.05$).

$k_1$: rate of glucose appearance in the gut.

$k_5$: rate of insulin-dependent glucose uptake to peripheral tissues.

$k_6$: rate of glucose-dependent insulin secretion.

*CGM* continuous glucose monitoring.

## Discussion

Physiology-based mathematical modeling of the glucose-insulin dynamics enables the quantification of key parameters, such as insulin sensitivity and insulin secretion^13^. However, their calibration depends on the availability of invasive measurements such as time-series of plasma glucose and insulin after an OGTT. Minimally-invasive, frequently-sampled interstitial glucose measurements from CGM devices have the potential to enable a broader impact of PBMMs by reducing their reliance on in-clinic measurements. Here, we presented a systematic comparison of using CGM glucose instead of plasma glucose in the calibration of the E-DES model by using simultaneously measured plasma and interstitial glucose measurements from an OGTT. We showed that the personalized E-DES models can fit the CGM profiles well and provide comparable parameter estimates of insulin secretion and insulin sensitivity when calibrated on interstitial glucose compared to plasma glucose concentrations after an OGTT in individuals with overweight or obesity. In addition, the associations between model parameters and the Matsuda and insulinogenic indices are also in agreement with our previous findings in a similar study population^17^. Differences in the models calibrated on interstitial compared to plasma glucose included a slightly lower estimate of the rate of glucose-dependent insulin secretion ($k_6$).

### Model fit and identifiability

In general, the personalized E-DES models displayed good agreement with the data both when calibrated on plasma glucose as well as CGM glucose in a wide range of glucose and insulin responses after an OGTT in individuals with overweight or obesity. The discrepancy in the MSE in simulation between glucose data types may originate in the difference in the proportion of available data for calibration between plasma and interstitial glucose. The more frequent sampling in the case of CGM glucose resulted in a lower MSE in the simulation of glucose, and a higher MSE in the simulation of insulin when compared to calibration on plasma glucose. More similar MSE between the glucose data types may be achieved by introducing a weighting of the glucose and insulin loss by number of available measurements in the objective function. However, the improvement in glucose MSE resulted in a comparatively small increase in insulin MSE (Fig. 2), therefore we chose to keep the original loss objective.

The heavily right skewed MSE distributions in models calibrated on plasma as well as CGM glucose suggest that there were only few personalized models that showed poor agreement with the data irrespective of glucose data type. Additionally, poor model fit (such as the ones in panel b, subpanels B, C in Fig. 3) was a result of a failed parameter search leading to a (upper) boundary value estimate for $k_6$. The boundary $k_6$ estimates were observed in only 16 personalized models in total, and more frequently when calibrating on plasma glucose (N=11) compared to CGM glucose (N=5). Coincidentally, the parameter $k_6$ was practically unidentifiable in these cases with the resulting models displaying large MSE in model fit. While this issue may be resolved by tuning the search bounds of the parameters, this should be done systematically, taking into account physiologically plausible regions of the parameter space. Alternatively, formulating the parameter estimation procedure in the Bayesian framework, relying on sampling the parameter space, may circumvent some of these issues^40,41^. However, exploring such avenues were outside the scope of the current study. Additionally, it is important to note, that while the MSE gives an overall indication of goodness of fit, it may be biased towards responses with higher measurement values irrespective of whether the temporal dynamics (a qualitative but important feature) are approximated well.

In addition to model fit, we evaluated the practical identifiability of each personalized model via profile likelihood analysis, to assess whether the quantity and quality of available experimental measurements are sufficient to have well-determined model parameters and predictions^38^. In total, 2% of the personalized models were found to be unidentifiable, suggesting that the model complexity is balanced for the availability of experimental data. Furthermore, due to the sampling frequency of the CGM device, approximately four times more glucose data was available when calibrating on CGM glucose compared to plasma glucose. Correspondingly, 6 personalized models were unidentifiable from CGM glucose compared to the 11 unidentifiable models from plasma glucose. This is in line with the expectation that more experimental data improves identifiability.

### Parameter estimates and personalized model-based representation of glucose regulation

No significant differences were found between the distribution of parameter estimates after calibration on plasma glucose compared to CGM glucose, except for $k_6$. This difference is driven by a handful of personalized models ending up with $k_6$ estimates on the upper boundary of the parameter search range. Excluding these models from the analysis, the discrepancy between plasma and CGM glucose-based $k_6$ estimates disappears. Nevertheless, out of the parameters of interest, $k_6$ showed the worst agreement after calibration between the glucose data types. This may indicate that a more complex model of the plasma-to-interstitium glucose equilibration is desired. However, increased similarity in model representation between the data types may not justify the added model complexity, especially when parameters are estimated from limited individual-specific experimental data. In addition, we found large inter-individual variability in lag time between interstitial and plasma glucose, consistent with earlier findings^42^. A more thorough inspection of the lag time between plasma and interstitium may be necessary to reliably characterize the inter-individual variability, ideally with experimental data containing repeated challenge tests in the same individuals.

Overall, the models showed good agreement between the glucose data types used in model calibration when comparing the parameter estimates with metabolic indicators. In particular, the parameter estimates of $k_5$, corresponding to insulin sensitivity, associated similarly with MISI, HOMA-IR, Matsuda index, M-value, HOMA-$\beta$ and the disposition index between models calibrated on plasma compared to CGM glucose. In the case of $k_1$ and $k_6$, representing glucose appearance in the gut, and rate of glucose-dependent insulin secretion, similar trends with differences in the strengths of the association with metabolic indicators were observed between the glucose data types. In particular, the differences in $k_1$ between plasma and CGM glucose are representative of the slower early phase (<30 min) dynamics in the case of CGM glucose. This is indicated by the discrepancy in the correlation of $k_1$ with metabolic indicators derived from the 0-30 min measurements, such as the $AUC^{30}$, the insulinogenic index or HIRI. Similarly, the differences in the associations of $k_6$ with metabolic indicators were also more prevalent in the case of indices targeting the early phase of the responses, such as HIRI and the disposition index. While, differences in the associations of $k_6$ with metabolic indicators such as MISI is indicative of the difference in the late phase of the glucose concentrations between plasma and CGM glucose. Finally, the difference between the $k_6$ estimates between the data types are indicative of the relative nature of the model-based rate of glucose-dependent insulin secretion ($k_6$). As the definition of the parameter suggests, the estimated rate of glucose-dependent insulin secretion is in the context of the glucose concentrations. Therefore the same insulin concentrations may be simulated with different rate parameters for insulin secretion given differences in glucose concentrations. This is reinforced by the weaker association of $k_6$ with the disposition index when estimated from interstitial glucose. The disposition index is a measure of $\beta$-cell function normalized to insulin sensitivity (approximated by the glucose area under the response curve). Since the interstitial glucose responses tended to be larger than plasma glucose responses, the normalization leads to comparatively lower disposition index.

### CGM data in dynamic models of glucose homeostasis

The potential use of CGM glucose in the calibration of a computational model describing glucose-insulin dynamics in type 1 diabetes (T1D) has previously been explored by Goel et al. (2018), however, in their study, insulin data was scarcely available^27^. Similarly, Eichenlaub et al. (2019), adapted the oral glucose minimal model to be calibrated on CGM glucose only, in healthy individuals, individuals with prediabetes, and patients with T2D and incorporated the effect of subsequent meals^12,28^. However, in order to accurately estimate the glucose-insulin dynamics in individuals with intact endogenous insulin secretion, the use of insulin (or insulin proxy) measurements in model calibration are crucial due to altered insulin secretion and insulin resistance^43,44^. In addition, changes in insulin secretion and its relation to circulating glucose levels in the development of both T1D and T2D have been reported to be non-monotonic, rendering the estimation of insulin secretion purely from glucose very challenging^45,46^. The importance of insulin in model calibration is also highlighted in an example simulation showcasing a model calibrated with and without insulin data (Fig. S2).

Recently, Ng et al. (2022), developed a parsimonious mathematical model of glucose homeostasis calibrated solely on peaks and nadirs extracted from CGM glucose^29^. Their three-parameter model showed better agreement with CGM glucose compared to other models including the Bergman minimal model^11^. However, in line with the previous studies, the utility of quantifying (disrupted) glucose regulation with PBMMs is limited when relying solely on glucose as the input. The present study also demonstrates the use of a parsimonious model with three estimable parameters, however, the use of both glucose and insulin data allows our model to infer various states of insulin resistance by accounting for $\beta$-cell adaptation. Similarly to our results, Faggionato et al. (2023) showed good agreement between the parameter estimates of the Oral Minimal Model when estimated with plasma compared to interstitial glucose in patients with T1D^33^.

There is a growing need to reduce the reliance on in-clinic measurements, and make use of free-living or at-home measurements such as CGM for the diagnosis and management of diabetes as well as in applications of precision nutrition^19^. Combining sensor data from wearable devices with mathematical modeling may serve as the basis for the development of digital twin technologies that enable *in silico* testing, disease prediction and optimization of treatment in a personalized setting^47–49^. While conventional plasma insulin measurements were available for model calibration in the current study, recent advances in dried blood spot analysis for determination of C-peptide present a promising alternative to the in-clinic measurements of C-peptide or insulin^50,51^. Furthermore, novel insulin sensors for non-invasive, real-time monitoring of bioavailable insulin are being actively developed^52^. The use of such insulin or C-peptide data in combination with CGM may allow for the calibration of glucose homeostasis models completely based on at-home measurements. In addition, the minimally invasive nature, and the higher sampling frequency (5-15 min) of CGM may support a comparatively sparse insulin sampling in turn, to facilitate model calibration. The parsimonious representation of an individual’s glucose homeostasis as given by the parameter estimates of such models may in turn enable the monitoring of changes in metabolic health.

However, there remain issues such as the CGM accuracy being dependent on glucose levels, and glucose rate-of-change that may influence modeling results^24,53^. Recently, Howard et al. (2020), reported inconsistencies between different glucose monitors worn simultaneously, while another group found good agreement between devices^54,55^. Systematic differences between data from different devices will affect model calibration and may hinder subsequent model-based analysis. In addition, the (dis)agreement of CGM-based interstitial glucose with plasma glucose should be considered when used to calibrate a model that was designed and developed to be used with plasma glucose^26,56,57^. Despite the addition of an interstitial compartment to account for the lag time between plasma and interstitial glucose concentrations, the dynamics between the data types may vary considerably. However, in order to accurately characterize this variability on the individual level, repeated challenges may be necessary in the same individuals to disentangle technical and biological sources of variation. In our study, the physiological representation of the personalized models showed good agreement between plasma glucose and interstitial glucose, however, the data used here originate from controlled conditions of an OGTT. Therefore, the similarity between the models estimated from the different data types observed in our work may not extend to other scenarios and populations.

## Conclusions

The use of interstitial glucose data from CGM was evaluated and compared with conventional plasma glucose from an OGTT in the calibration of personalized E-DES models in individuals with overweight or obesity. Results indicated comparable model fits, and parameter estimates between CGM and plasma glucose. A slightly lower estimate of insulin secretion was observed in the case of CGM glucose, due to the difference in dynamics compared to plasma glucose. Nonetheless, the model-based measure of insulin sensitivity and glucose-dependent insulin secretion were validated by well-established measures of metabolic health such as the Matsuda index and the insulinogenic index after calibration on both data types. Finally, more personalized models were found to be practically identifiable when calibrated on CGM glucose compared to plasma glucose, likely due to the higher sampling frequency of CGM. The use of non-invasive CGM data in dynamic modeling of the glucose-insulin system has the potential to advance precision nutrition as well as diabetes prevention and management. However, it is important to note that our findings are derived from data obtained through a conventional OGTT including plasma insulin measurements. Therefore, the applicability of these results to data from less controlled experimental settings may be limited. Finally, care must be taken when replacing plasma glucose measurements with CGM glucose in PBMMs as it may influence model interpretation.

## Supplementary Information

Supplementary Information.

## Data Availability

Data from the PERSON Study are unsuitable for public deposition due to ethical restriction and privacy of participant data. Data are available to researchers meeting the criteria for access to confidential data. Gabby Hul on behalf of the data management team of the department of Human Biology may be contacted at g.hul@maastrichtuniversity.nl to request the PERSON Study data.
