# Supplementary material for: Leveraging continuous glucose monitoring for personalized modeling of insulin-regulated glucose metabolism
Source: Sci Rep. 2024 Apr 5;14:8037. doi: 10.1038/s41598-024-58703-6 (PMC11371931; doi:10.1038/s41598-024-58703-6)
Supplement: Supplementary file 1 — Supplementary Information. [file 41598_2024_58703_MOESM1_ESM.pdf]

Supplementary Material

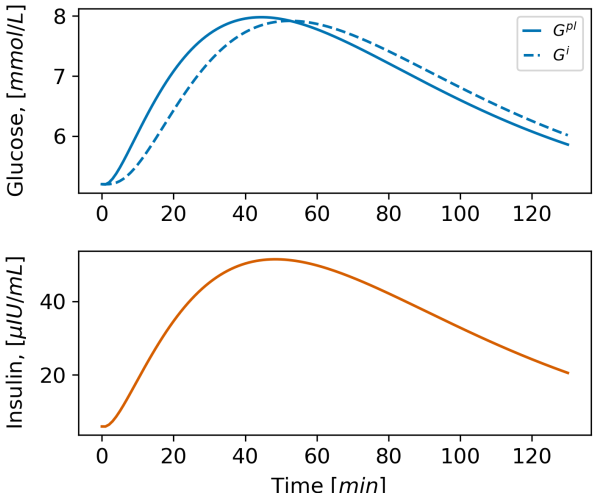

Supplementary Figure S1. EDES model simulated plasma glucose and interstitial glucose concentration (continuous and dashed lines, respectively; top panel), as well as plasma insulin concentration (bottom panel). In order to account for the delay in plasma to interstitium, a linear compartment was introduced to the EDES model as described in [1], [2].

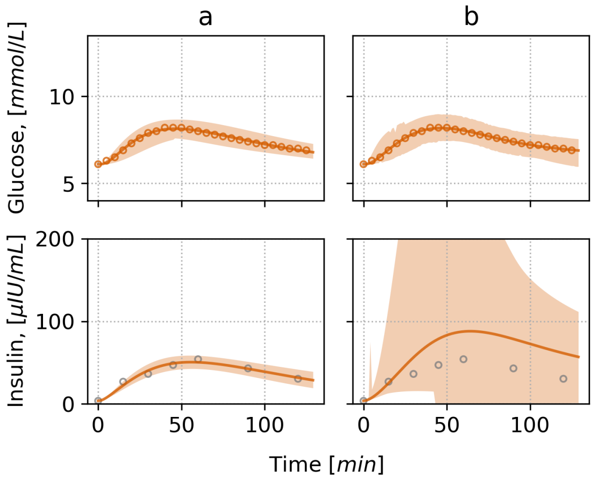

Supplementary Figure S2. Example of a simulated OGTT response from the EDES model after calibration on interstitial glucose and plasma insulin (panel a) compared to calibration on interstitial glucose only (panel b). Model simulation is depicted by continuous line while the data used in calibration are denoted by points. Note that only the interstitial glucose was used in calibration before simulating the response on panel b highlighting the need for insulin measurements for an accurate representation of the response. The shaded regions represent a confidence band derived by estimating 95% confidence intervals of the observables as a function of all estimated parameters over time.

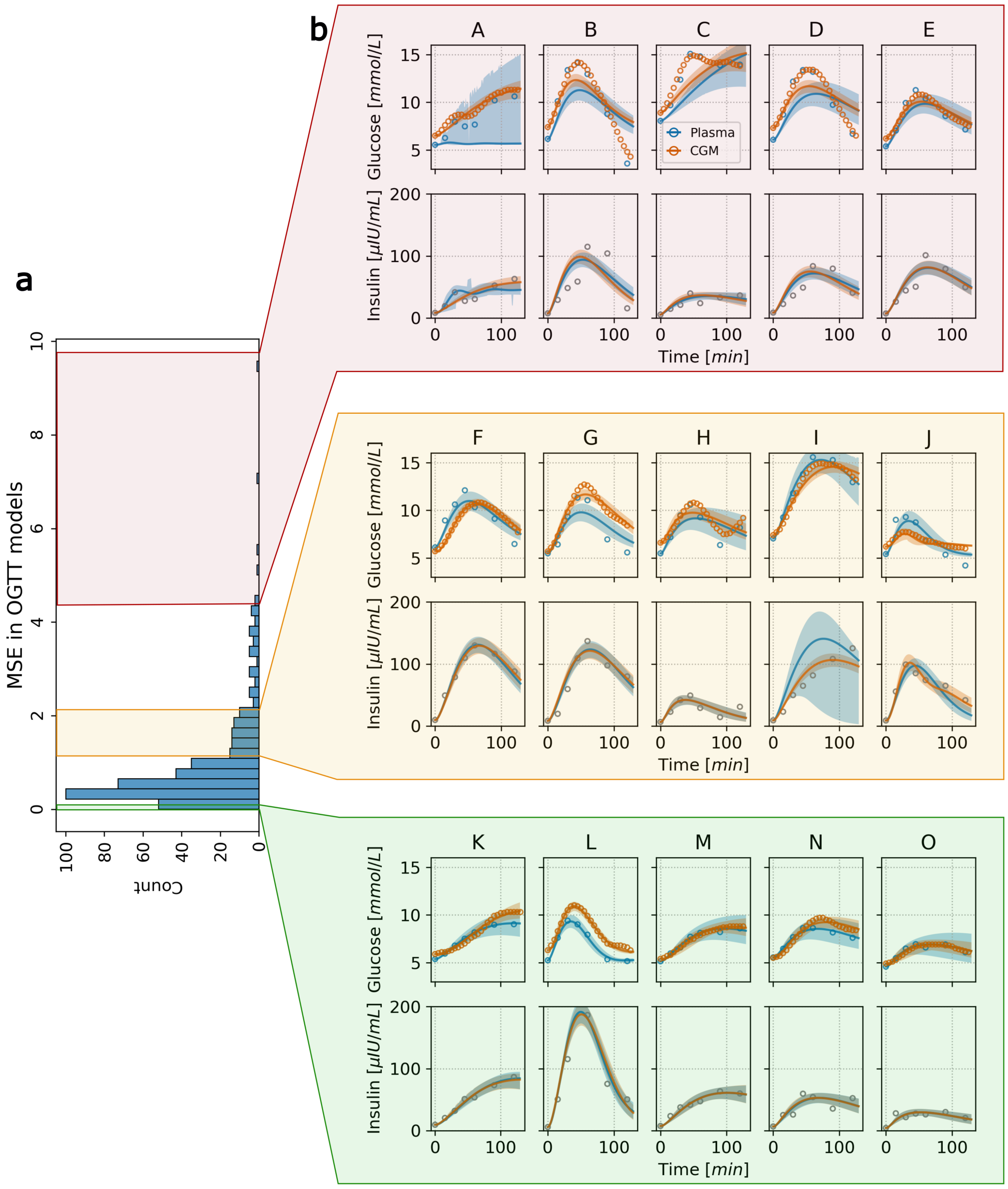

Supplementary Figure S3. Model fit examples of personalized E-DES models arranged by MSE in the simulation of plasma glucose. Panel a: histogram of MSE in glucose simulation. The three sections with red, yellow, and green shading indicate models with the worst, examples around the mean, and best fit, respectively, as measured by the MSE. Panel b: Glucose and insulin data of participants are shown as circles (A-O) with corresponding model simulation shown as continuous line. Blue and orange color indicates the type of glucose measurement (plasma vs CGM glucose), and simulations from the correspondingly calibrated models. The shaded region around the simulation corresponds to confidence bands generated from estimated confidence intervals (with confidence level 0.95) as function of all parameters over simulation time-points. CGM: continuous glucose monitoring, MSE: mean squared error

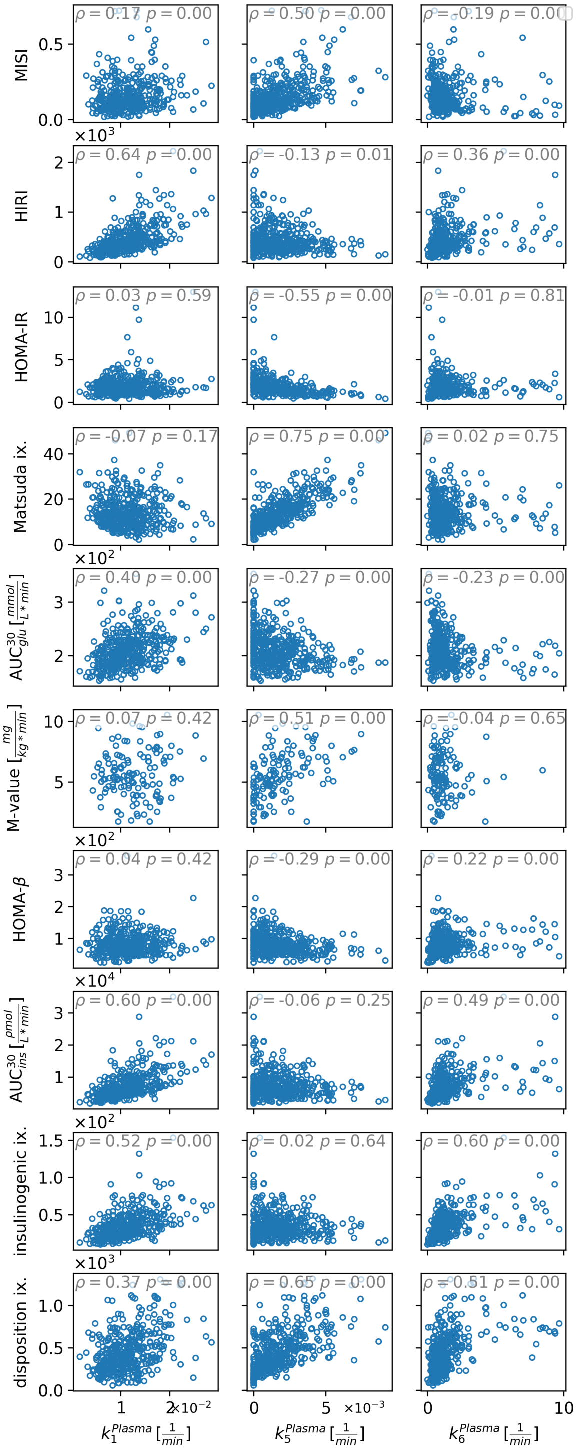

Supplementary Figure S4. Association of parameter estimates from CGM glucose with metabolic indicators. k_1: rate of glucose appearance in the gut, k_5: rate of insulin-dependent glucose uptake to peripheral tissues, and k_6: rate of insulin secretion. OGTT: oral glucose tolerance test, CGM: continuous glucose monitoring.

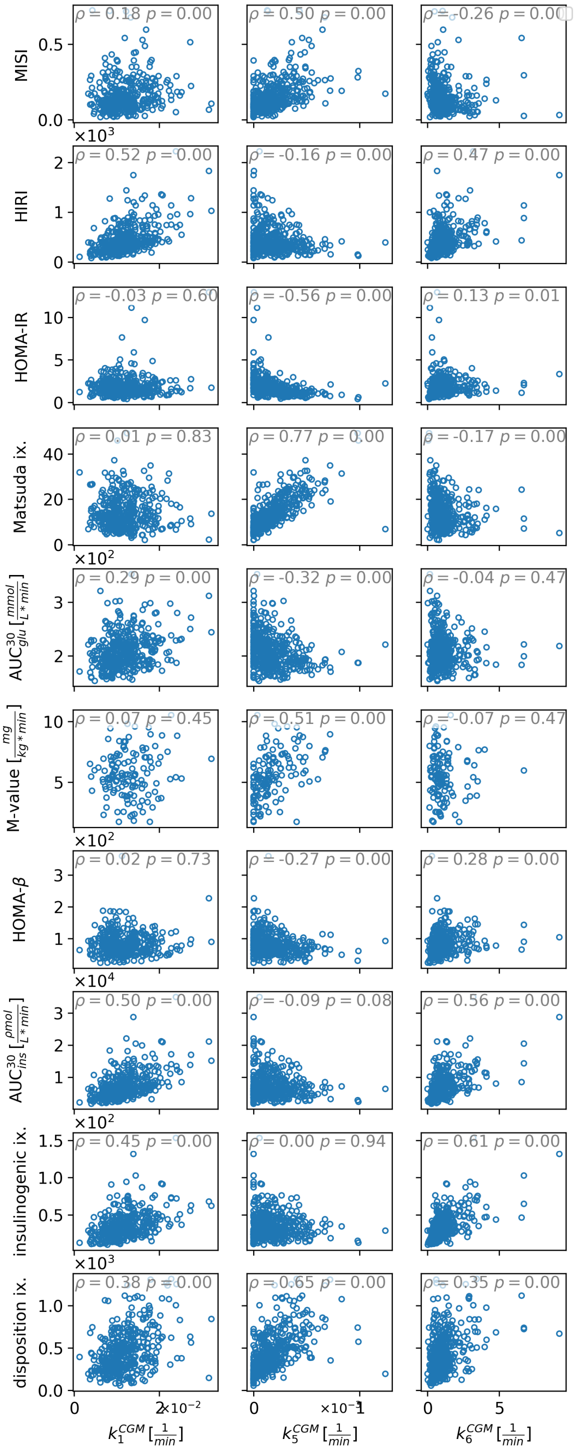

Supplementary Figure S5. Association of parameter estimates from plasma glucose with metabolic indicators. k_1: rate of glucose appearance in the gut, k_5: rate of insulin-dependent glucose uptake to peripheral tissues, and k_6: rate of insulin secretion. OGTT: oral glucose tolerance test, CGM: continuous glucose monitoring.

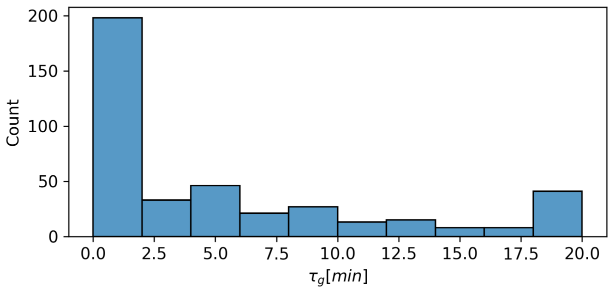

Supplementary Figure S6. Histogram of τ_g estimates from model calibration on individual specific CGM glucose and plasma insulin.

Supplementary Appendix

E-DES model structure, fluxes, inputs, parameters and constants. Adapted from Maas et al., 2015. The model was extended with a linear interstitial compartment to account for the delay from plasma to insterstitium as previously described in [1], [2].

Glucose in the gut

$$\frac{dM_G^{gut}}{dt} = m_G^{meal}(D^{meal}, t) - m_G^{pl}(M_G^{gut}) \tag{1}$$

$$m_G^{meal} = \sigma k_1^{\sigma} t^{\sigma-1} exp(-(k_1 t)^{\sigma}) D^{meal} \tag{2}$$

$$m_G^{pl} = k_2 M_G^{gut} \tag{3}$$

Glucose in the plasma

$$\frac{dG^{pl}}{dt} = g^{liv}(G^{pl}, I^{pl}) + g^{gut}(M_G^{gut}) - g^{non-it}(G^{pl}) - g^{it}(G^{pl}, I^{pl}) - g^{ren}(G^{pl}) \tag{4}$$

$$g^{liv} = g_b^{liv} - k_3(G^{pl} - G_b^{pl}) - k_4\beta(I^{pl} - I_b^{pl}) \tag{5}$$

$$g^{gut} = \frac{f}{V_G M^b} m_G^{pl} = k_2 \frac{f}{V_G M^b} M_G^{gut} \tag{6}$$

$$c_2 = g_b^{liv}\left(\frac{K_M + G_b^{pl}}{G_b^{pl}}\right) \tag{7}$$

$$g^{non-it} = c_2 \frac{G^{pl}}{K_M + G^{pl}} \tag{8}$$

$$g^{it} = k_5 \beta I^{pl} \frac{G^{pl}}{K_M + G^{pl}} \tag{9}$$

$$g^{ren} = \begin{cases} \frac{c_1}{V_G M^b}(G^{pl} - G_{th}^{pl}), & \text{if } G^{pl} > G_{th}^{pl} \quad (10) \\ 0, & \text{if } G^{pl} \leq G_{th}^{pl} \quad (11) \end{cases}$$

Glucose in the interstitium

$$\frac{dG^i}{dt} = \frac{1}{\tau_g}(G^{pl} - G^i) \tag{12}$$

Insulin in the plasma

$$\frac{dI^{pl}}{dt} = i^{pnc}(G^{pl}) - i^{liv}(I^{pl}) - i^{if}(I^{pl}) \tag{13}$$

$$i^{pnc} = \beta^{-1}\left(k_6(G^{pl} - G_b^{pl}) + \left(\frac{k_7}{\tau_i}\right) G_b^{pl} + (k_8 \tau_d)\frac{dG^{pl}}{dt}\right) \tag{14}$$

$$c_3 = k_7 \frac{G_b^{pl}}{\beta \tau_i I_b^{pl}} \tag{15}$$

$$i^{liv} = c_3 I^{pl} \tag{16}$$

$$i^{if} = k_9(I^{pl} - I_b^{pl}) \tag{17}$$

Overview of the E-DES model (input) variables

Name	Description	Units
t	Time	min
$M_G^{gut}(t)$	Glucose mass in the gut	mg
$G^{pl}(t)$	Plasma glucose concentration	mmol/L
$I^{pl}(t)$	Plasma insulin concentration	mU/L
$G^i(t)$	Interstitial glucose concentration	mmol/L
D^{meal}	Glucose intake	mg
M^b	Body mass	kg

In the current study, glucose intake was set to 75g and body mass was set to 70kg.

Overview of the E-DES model fluxes

Name	Description	Units
$m_G^{meal}(t)$	Glucose mass entering from stomach	mg/min
$m_G^{pl}(t)$	Glucose mass leaving to plasma	mg/min
$g^{liv}(t)$	Glucose production by the liver (EGP)	mmol/L/min
$g^{gut}(t)$	Glucose entering from the gut	mmol/L/min
$g^{non-it}(t)$	Glucose uptake by insulin-independent tissue	mmol/L/min
$g^{it}(t)$	Glucose uptake by insulin-dependent tissue	mmol/L/min
$g^{ren}(t)$	Renal glucose elimination	mmol/L/min
$i^{pnc}(t)$	Pancreas insulin secretion	mU/L/min
$i^{if}(t)$	Insulin flowing into interstitial fluid	mU/L/min
$i^{liv}(t)$	Insulin uptake by the liver	mU/L/min

Overview of the E-DES model parameters

Name	Description	Units	Value
$k1$	Rate constant of glucose appearance in the gut	1/min	1.35e-2
$k2$	Rate constant of gut emptying	1/min	6.33e-1
$k3$	Rate constant of ΔG suppression of EGP when $G^{pl} > G_b$	1/min	5.00e-5
$k4$	Rate constant of insulin-dependent suppression of EGP	1/min	1.00e-3
$k5$	Rate constant of insulin-dependent glucose uptake	1/min	3.80e-3
$k6$	Rate constant of ΔG dependant insulin production	1/min	5.82e-1
$k7$	Rate constant of $\int G$ dependant insulin production	1/min	1.15
$k8$	Rate constant of $\frac{dG}{dt}$ dependant insulin production	1/min	4.71
$k9$	Rate constant of insulin outflow from plasma to interstitial fluid	1/min	1.08e-2
σ	Shape factor	-	1.35
K_M	Michaelis-Menten constant for glucose uptake	mmol/L	0.63
τ_g	Equilibration time constant between plasma and interstitium	min	2.5*

Parameter values from Maas, 2017.
* Estimated from experimental data.

Overview of the E-DES model constants

Name	Description	Units	Value
G_b^{pl}	Basal plasma glucose *	mmol/L	$G^{pl}(t=0)$
I_b^{pl}	Basal plasma Insulin *	mU/L	$I^{pl}(t=0)$
g_b^{liv}	Basal endogenous glucose production	mmol/L/min	0.043
G_{th}^{pl}	Renal threshold	mmol/L	9
V_G	Glucose distribution volume in plasma	L/kg	17/70
β	Unit conversion factor from glucose to insulin	(mmol/L)/(mU/L)	1
f	Unit conversion factor from mmol to mg glucose	mmol/mg	0.005551
τ_i	Integral time constant	min	31
τ_d	Derivative time constant	min	3
$c1$	Rate constant of glomerular filtration	1/min	0.1
$c2$	Rate constant of glucose uptake by non-insulin dependent tissue	1/min	-
$c3$	Rate constant of liver insulin clearance	1/min	-

Constant values from Maas, 2017.
* From experimental data

Parameter boundaries in optimization and scan bounds of profile likelihood analysis

		Parameter search		Profile scan	
Parameter	Default*	Lower bound	Upper bound	Lower bound	Upper bound
k_1	$1.35e-2$	$1e-7$	$3e-1$	$1.0e-10$	Default × $3.0e2$
k_5	$3.80e-3$	$1e-7$	1.0	$1.0e-10$	Default × $1.0e2$
k_6	$5.82e-1$	$1e-7$	$1.0e1$	$1.0e-10$	Default × $1.0e2$
τ_g	2.5	$1e-10$	20	-	-

* The default parameter values are used as initial values to the TikTak algorithm in the optimization.

Calculation of metabolic indicators

The Matsuda index was calculated using glucose [mg/dL] and insulin [mU/L] values of time points 0, 30, 60, 90, 120 min of the OGTT as discussed in [3]. The disposition index was calculated as [Matsuda index * ($AUC_{ins}^{30}/AUC_{glu}^{30}$)], where AUC^{30} is the area under the curve between baseline and 30 min of the OGTT for insulin [$\rho mol/L$] and glucose [$mmol/L$], respectively, as calculated by the trapezoidal method. The homeostasis model assessment of insulin resistance (HOMA-IR) was calculated using fasting glucose [$mmol/L$] and fasting insulin [mU/L] according to [4]. HOMA of β-cell function (HOMA-β) was calculated as (20 $\times$ fasting insulin [mU/L])/(fasting glucose [$mmol/L$] – 3.5). Insulinogenic index was calculated as ((insulin^{30min}-insulin^{0min})/(glucose^{30min}-glucose^{0min})). The muscle insulin sensitivity index (MISI) was calculated as (dGlucose/dt)/(mean insulin [$\rho mol/L$], where dGlcuose/dt is the rate of decay of plasma glucose [$mmol/L$] during the OGTTm cakcykated as described in [5]. The hepatic insulin resistance index (HIRI) was calculated as ($AUC_{glu}^{30} \times AUC_{ins}^{30}$) where AUC^{30} is the area under the curve between baseline and 30 min of the OGTT for glucose [$mmol/L$] and insulin [$\rho mol/L$], respectively. Finally, the M-value representing peripheral insulin sensitivity was derived after a 2.5h two-step hyperinsulinemic-euglycemic clamp with constant 40 $mU/m^2/min$ infusion of insulin [6].

References

[1] K. Rebrin, G. M. Steil, W. P. van Antwerp, and J. J. Mastrototaro, "Subcutaneous glucose predicts plasma glucose independent of insulin: Implications for continuous monitoring," *American Journal of Physiology-Endocrinology and Metabolism*, vol. 277, no. 3, E561–E571, Sep. 1999, Publisher: American Physiological Society. DOI: 10.1152/ajpendo.1999.277.3.E561.

[2] E. Faggionato, M. Schiavon, L. Ekhlaspour, B. A. Buckingham, and C. Dalla Man, "The minimally-invasive oral glucose minimal model: Estimation of gastric retention, glucose rate of appearance, and insulin sensitivity from type 1 diabetes data collected in real-life conditions," *IEEE Transactions on Biomedical Engineering*, pp. 1–9, 2023. DOI: 10.1109/TBME.2023.3324206.

[3] M. Matsuda and R. A. DeFronzo, "Insulin sensitivity indices obtained from oral glucose tolerance testing: comparison with the euglycemic insulin clamp.," *Diabetes Care*, vol. 22, no. 9, pp. 1462–1470, Sep. 1999. DOI: 10.2337/diacare.22.9.1462.

[4] D. R. Matthews, J. P. Hosker, A. S. Rudenski, B. A. Naylor, D. F. Treacher, and R. C. Turner, "Homeostasis model assessment: Insulin resistance and β-cell function from fasting plasma glucose and insulin concentrations in man," *Diabetologia*, vol. 28, no. 7, pp. 412–419, Jul. 1985. DOI: 10.1007/BF00280883.

[5] S. D. O'Donovan, M. Lenz, G. H. Goossens, C. J. H. van der Kallen, S. J. M. P. Eussen, C. D. A. Stehouwer, M. M. van Greevenbroek, M. T. Schram, S. J. Sep, R. L. M. Peeters, E. E. Blaak, N. A. W. van Riel, T. M. C. M. de Kok, and I. C. W. Arts, "Improved quantification of muscle insulin sensitivity using oral glucose tolerance test data: The MISI Calculator," *Scientific Reports*, vol. 9, no. 1, p. 9388, Jun. 2019. DOI: 10.1038/s41598-019-45858-w.

[6] R. A. DeFronzo, J. D. Tobin, and R. Andres, "Glucose clamp technique: A method for quantifying insulin secretion and resistance.," *American Journal of Physiology-Endocrinology and Metabolism*, vol. 237, no. 3, E214, 1979. DOI: 10.1152/ajpendo.1979.237.3.E214.
